# ASAS-NANP SYMPOSIUM: Mathematical Modeling in Animal Nutrition: Training the Future Generation in Data and Predictive Analytics for Sustainable Development. A Summary of the 2023 Symposium

**DOI:** 10.1093/jas/skaf141

**Published:** 2025-05-04

**Authors:** Luis O Tedeschi, Hector M Menendez

**Affiliations:** Texas A&M University, Department of Animal Science, College Station, TX 77843-2471, USA; South Dakota State University West River Research and Extension Center, 711 N. Creek Dr. Rapid City, SD, 57701, USA

## INTRODUCTION

Mathematical modeling and data analytics are two critical components for achieving sustainability in livestock production. While scientific knowledge remains the most crucial step in model-building (Tedeschi, 2023), educating and training students and researchers in modeling techniques are essential first steps to developing the necessary discipline and skills for successful mathematical model development (Ellis et al., 2020; Tedeschi, 2019). Due to limited opportunities for students and researchers to understand the underlying principles of mathematical modeling, the Modeling Committee of the National Animal Nutrition Program (**NANP**; https://animalnutrition.org) was tasked with enhancing modeling skills for future generations in animal science programs by providing training venues for quantitative and qualitative modeling approaches to data and predictive analytics.

Over the past seven years (2018 to 2024), these workshops have consistently demonstrated their significant value and impact on the field. Their success is evidenced by a steady attendance of 60 to 100 participants annually, drawing a diverse audience of graduate students, faculty, and industry professionals—a clear indication of the sustained demand for advanced training in mathematical modeling within the animal nutrition community. Surveys conducted across four symposia (2020, 2021, 2022, and 2023) highlighted the broad diversity and depth of modeling knowledge among participants from academia, industry, and government sectors. These surveys have consistently revealed high satisfaction rates, with many attendees indicating they would recommend the workshops to colleagues. Additionally, participants expressed a strong interest in expanding their knowledge of various modeling techniques. The scholarly impact of these symposia is further demonstrated by the peer-reviewed publications they’ve generated, which have been cited more than 250 times, indicating their substantial contribution as valuable resources for colleagues in their research efforts.

## THE SYMPOSIA

The Modeling Committee of the National Research Support Project #9 from the NANP has organized six symposia under the theme “*Mathematical Modeling in Animal Nutrition: Training the Future Generation in Data and Predictive Analytics for Sustainable Development*” in association with the American Society of Animal Science (**ASAS**), American Society for Nutrition (**ASN**), Canadian Society of Animal Science (**CSAS**), Southern Section of the American Society of Animal Science (**SSASAS**), or Western Section of the American Society of Animal Science (**WSASAS**). All presentations are available at https://animalnutrition.org/workshops-symposia.

The first symposium, “*The Future of Livestock Research: Knowledge Gaps, Data Collection and Quality, and the Role of Supporting Tools for Sustainable Production,*” was held in Vancouver, Canada, on July 8, 2018, featuring primarily 1.5-hour hands-on training workshops. The second symposium, “*Mathematical Model Building and Evaluation, and Data Analyses,*” took place in Austin, TX, on July 8, 2019, and consisted of five 50-minute presentations. These two events generated two publications due to their similar topics (Nicholson et al., 2019; Tedeschi, 2019). The third symposium, held virtually on July 19, 2020, under the title “*Mathematical Modeling in Animal Nutrition: Training the Future Generation in Data and Predictive Analytics for a Sustainable Development,*” yielded four publications (Gerrits et al., 2021; Morota et al., 2021; Stephens, 2021; Wang et al., 2021). The fourth symposium in Louisville, KY, on July 14, 2021, produced three publications (Jacobs et al., 2022; Menendez et al., 2022; Tedeschi, 2022), while the fifth symposium in Oklahoma City, OK, on June 26, 2022, resulted in three additional publications (Brennan et al., 2023; Kaniyamattam and Tedeschi, 2023; Muñoz-Tamayo and Tedeschi, 2023). Comprehensive descriptions of these 12 publications were provided by Tedeschi et al. (2021) and Tedeschi et al. (2023).

The sixth symposium in Albuquerque, NM, on July 16, 2023, featured three 40-minute presentations and three 1.5-hour hands-on training workshops, creating a balanced learning environment that addressed theoretical concepts and practical applications in mathematical modeling for animal nutrition. It comprehensively explored data processing challenges and decision support systems, with lectures on real-time data integration, satellite-based tools for grazing cattle production, and poultry modeling evolution. Hands-on sessions focused on agent-based modeling (**ABM**) in AnyLogic, system dynamics (**SD**) modeling for sustainable livestock production, and digital twin development for precision livestock farming (**PLF**), with special attention to data analytics and big data challenges. Tedeschi (2025) introduced a novel rank-based method for generating synthetic databases with correlated non-normal multivariate distributions to address the challenge of limited data availability in animal science, particularly for modeling complex biological processes like methane emissions from ruminants. This research addresses a critical challenge in agricultural data science—the scarcity of comprehensive datasets for developing artificial intelligence (**AI**) oriented models. This approach could help bridge the gap between traditional mechanistic models and modern AI applications, supporting the development of hybrid intelligent mechanistic models (**HIMM**) that describe underlying principles while handling massive amounts of data. Fernandes and Tedeschi (2025) showed the integration of satellite remote sensing technology with mathematical modeling to enhance sustainable grazing cattle management. The paper categorizes modeling approaches into parametric regression models (based on vegetation indices), nonparametric regression models (including machine learning (**ML**) algorithms), and physically based models. They highlighted significant advancements in satellite technology, particularly the launch of Sentinel-2, which provides improved spectral, spatial, and temporal resolution for monitoring pasture parameters. They also note the growing adoption of ML algorithms since 2020 for estimating above-ground biomass and forage nutritional attributes. The paper discusses practical applications integrating satellite-derived vegetation data with animal nutrition models, demonstrating how these technologies can support real-time grazing management by adjusting stocking rates and predicting average daily gain. Oviedo-Rondón (2025) overviewed the poultry nutrition modeling, tracing its evolution over the past 45 years. The paper discussed two major mechanistic modeling systems, the EFG (Emmans, Fisher, and Gous) and AVINESP models, explaining their theoretical frameworks for describing growth, egg production, and nutrient requirements. It details how these models partition energy requirements, estimate amino acid needs, and predict feed intake under various conditions. The author emphasized the potential for improving model accuracy by integrating new technologies such as near-infrared spectroscopy, bioelectrical impedance, sensor-based technologies, and system-wide multi-omics. Brown-Brandl and Tao (2025) examined the role of real-time data and digital twins in PLF, emphasizing their potential to enhance animal management and sustainability. The authors discussed key PLF technologies, including image-based monitoring, sound analysis, RFID tracking, and wearable sensors. They highlighted how digital twins integrate these data sources to create automated and individualized management systems. While promising, adopting digital twins faces challenges such as cost, data security, and the need for interdisciplinary collaboration. The paper envisioned AI, blockchain, and augmented reality enhancing PLF and digital twin applications. Integrating real-time data with digital twins can drive sustainability, optimize resource use, and transform livestock farming into a more efficient and precise industry.

These manuscripts demonstrated the evolution of agricultural models from theoretical exercises to practical decision-making tools, emphasizing mechanistic approaches that better represent biological processes and system dynamics. All papers highlighted the integration of traditional modeling with ML: Tedeschi (2025) compared random forest with linear models, Fernandes and Tedeschi (2025) documented ML adoption in satellite data analysis, and Oviedo-Rondón (2025) referenced neural networks for prediction enhancement. Brown-Brandl and Tao (2025) further expanded this integration by examining real-time data and digital twins in precision livestock farming, showing how these technologies create automated, individualized management systems despite adoption challenges. Environmental sustainability emerged as a unifying theme across all works, addressing methane emissions, grazing management, and nutrient efficiency in poultry production. The field increasingly adopts hybrid approaches combining complementary methods: mechanistic models with AI, satellite data with nutritional models, and conventional modeling with emerging multi-omics technologies. This hybrid modeling trend developed gradually through a series of symposia spanning from 2018 to 2023.

The evolution of mathematical modeling in animal nutrition began with foundational concepts in early symposia (2018-2019). Nicholson et al. (2019) emphasized system dynamics modeling to link system structure with dynamic behavior, while Tedeschi (2019) outlined core modeling terminology and noted scientists’ hesitancy toward systems thinking. A significant transition occurred in 2020-2021 when AI emerged as a central focus, addressing the “shiny object syndrome” while exploring practical applications in animal science. This period saw the conceptualization of HIMM and systematic implementation frameworks, including Menendez et al.’s (2022) five-step process for implementing PLF. By 2022, the focus shifted to specialized methodological aspects—identifiability analysis, agent-based modeling, and AI integration with individual animal data—while emphasizing data sharing and open-source platforms. This progression from theoretical foundations to practical applications validated Tedeschi’s (2019) prediction that “the successful future of mathematical modeling (**MM**) might rely on the development of redesigned models that can integrate existing technological advancements in data analytics.” The consistent attendance and citation rates of resulting publications demonstrate the field’s vitality and ongoing need for advanced modeling training.

The 2023 symposium papers illustrated this evolution’s culmination in practical applications across methane prediction, grazing management, poultry nutrition, and digital twin technology. Future advancements will require better integration of real-time data technologies with existing models, more user-friendly interfaces, and hybrid approaches leveraging both ML and mechanistic principles. Integrating these diverse streams will be crucial for sustainable animal production as data collection expands through sensors, satellite imagery, and -omics technologies. Emerging natural language processing could transform researcher-model interactions, making sophisticated analytical tools more accessible and enhancing model interpretability for broader adoption in practical animal nutrition applications.

## Abbreviations

ABM: agent-based model/modeling
AI: artificial intelligence
ASAS: American Society of Animal Science
ASN: American Society for Nutrition
CSAS: Canadian Society of Animal Science
HIMM: hybrid intelligent mechanistic models
ML: machine learning
NANP: National Animal Nutrition Program
PLF: precision livestock farming
SD: system dynamics
SSASAS: Southern Section of the American Society of Animal Science
and WSASAS: Western Section of the American Society of Animal Science
